# Dose decision of HSK7653 oral immediate release tablets in specific populations clinical trials based on mechanistic physiologically-based pharmacokinetic model

**DOI:** 10.1016/j.ejps.2023.106553

**Published:** 2023-10-01

**Authors:** Miao Zhang, Shudong Zhang, Zhiheng Yu, Xueting Yao, Zihan Lei, Pangke Yan, Nan Wu, Xu Wang, Qin Hu, Dongyang Liu

**Affiliations:** a Drug Clinical Trial Center, Peking University Third Hospital, Beijing, China; b Department of Pharmaceutical Sciences, School of Pharmacy and Pharmaceutical Sciences, University at Buffalo, The State University of New York, Buffalo, NY, United States; c Beijing Institute for Drug Control, NMPA Key Laboratory for Research and Evaluation of Generic Drugs, Beijing Key Laboratory of Analysis and Evaluation on Chinese Medicine, Beijing, China; d Department of Obstetrics and Gynecology, Peking University Third Hospital, Beijing, China; e Haisco Pharmaceutical Group Co., Ltd., Chengdu, China

**Keywords:** HSK7653 PBPK model, Systemic exposure, Specific population, Food effect

## Abstract

HSK7653, an oral dipeptidyl peptidase-4 inhibitor administered every 2 weeks, is a candidate for the treatment of type 2 diabetes mellitus. The major elimination pathway of HSK7653 *in vivo* is renal excretion, and hepatic metabolism and fecal excretion of unchanged compound contribute less to the systemic clearance of HSK7653. Considering the disposition characteristics and the potential indication population of HSK7653, evaluating the HSK7653 exposure in patients with renal impairment and geriatric populations is a prerequisite for bringing more benefits to the patients. Here, a PBPK model was developed based on *in vitro* experimental results, such as dissolution, permeability, and metabolism, and the *in vivo* renal clearance, to evaluate the effects of physiological factors and food on HSK7653 exposure in specific populations, including adult and elder individuals with renal impairment and geriatric populations. Simulation results showed that the AUC of HSK7653 increased by 46%, 82%, and 129% in adult patients with mild, moderate, and severe renal impairment, and by 56%, 78%, and 101% in patients aged 65–75, 75–85 and 85–95 years, respectively. The AUC increased in the range of 62%–83%, 98%–133%, and 153%–195% in elderly patients (65–95 years) with mild, moderate, and severe renal impairment, respectively. Moreover, two different absorption model development methods (dissolution profile method and the diffusion layer model method) predicted that food had no effect on the exposure of the same simulated population. Since the predicted AUC of HSK7653 at the 10 mg dose in various specific populations was still within the relatively flat results of the exposure-response analysis, the 10 mg dose of HSK7653 was first used to explore the exposure in the renal impairment population (CTR20221952).

## Highlights

• Dose decision of HSK7653 tablets in clinical trial of specific populations was determined according to the PBPK model prediction results.
• Meanwhile, the predictive performance of two model development methods on food effect was compared.
• Dissolution profile and Wang methods can be used to capture the absorption characteristics of compound without food effect *in vivo*, but the predictive accuracy of the former method is random.
• Only the latter method can be used to predict the mechanistic absorption characteristic of compound *in vivo*.

## Graphical abstract

**Figure:**
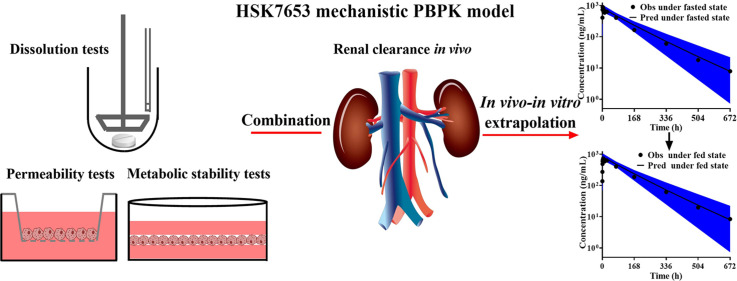

## 1 Introduction

Type 2 diabetes mellitus (T2DM) is a chronic and progressive disease with impaired glucose regulation, mainly caused by insulin resistance and progressive β-cell failure (Defronzo, 2009; Halban et al., 2014). In patients with T2DM, pharmacologic intervention is necessary to maintain normal glycemia levels and avoid complications. Many mechanisms of action can be used to correct the multiple pathophysiological defects and reverse pathogenic abnormalities (Defronzo, 2009). Researchers are working together to identify an ideal diabetes drug that can control glycemia without increasing the risk of hypoglycemia, improve pancreatic islet function, lose body weight and reduce other general risks (Wu et al., 2019). The contribution of incretin hormones, especially glucagon-like peptide-1 (GLP-1) (Vilsbøll et al., 2003), to the total insulin responses of healthy volunteer and T2DM patients is about 70% and 36% (Nauck et al., 1986), respectively. Thus, enhancing incretin action by providing physiological dosages of GLP-1 through GLP-1 receptor agonists or dipeptidyl peptidase-4 (DPP-4) inhibitors (incretin enhancers) is an effective treatment for patients with T2DM (Drucker and Nauck, 2006; Gilbert and Pratley, 2020). DPP-4 inhibitors can slow the degradation of GLP-1 and increase levels and prolong the action of GLP-1 (Deacon, 2020), resulting in the stimulation of insulin, suppression of glucagon secretion (Komatsu et al., 1989; Willms et al., 1996), and improvement in the islet cell function and lipid metabolism (Ahrén and Foley, 2016). For these reasons, various types of DPP-4 inhibitors are being actively investigated to conquer pancreatic islet dysfunctions.

**Fig. 1:**
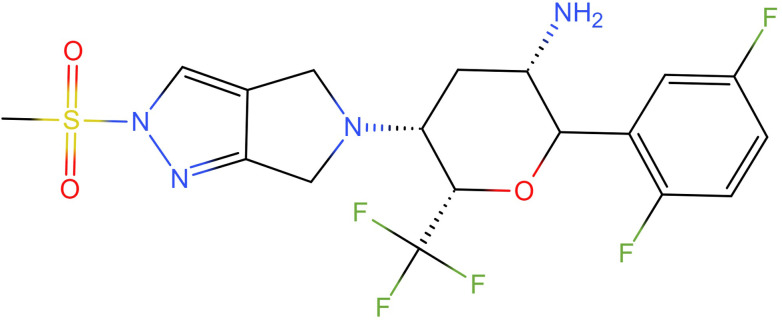
The chemical structure of HSK7653.

Haisco Pharmaceutical Group Co., Ltd. has developed an oral DPP-4 inhibitor HSK7653 administered every 2 weeks (structural formula shown in Fig. 1) with independent intellectual property rights (Zhang et al., 2020). The single ascending dose (SAD) clinical study of HSK7653 in Chinese healthy volunteers (CTR20180791), pharmacokinetic (PK) drug-drug interaction between HSK7653 and metformin (CTR20191549), and preliminary efficacy exploration studies of HSK7653 *in vivo* (CTR20182505, CTR20201759, and CTR20210174) have been completed with the good safety and tolerance. Meanwhile, in a multidose exploratory clinical study of T2DM patients (CTR20182505) the average inhibition rate of DPP-4 reached 80%, and the glycosylated hemoglobin was significantly reduced after six doses of oral administration, suggesting that HSK7653 has a significant effect on T2DM patients.

HSK7653 was found to be dose linear within the currently selected therapeutic dose range (CTR20180791). In the multidose clinical study of biweekly administration of HSK7653 immediate release (IR) tablets (CTR20182505), the blood drug concentration tended to be stable on the 15th day, and there was no accumulation in T2DM patients. The mass balance study (CTR20201559) showed that unchanged drug excreted by the kidney (68.1%) was the major elimination pathway, and hepatic metabolism (9.66% of metabolites in urine samples and 5.706% of metabolites in fecal samples) and fecal excretion of unchanged compound (7.40%) contributed less to the systemic clearance. Preclinical results demonstrated that HSK7653 was not a common transport substrate distributed in kidney (Ivanyuk et al., 2017), such as organic anions/cation transport protein (OATP1B1, OAT1, OAT3, OCT2, and OCT3), breast cancer resistance protein, multidrug and toxic compound extrusion proteins (MATE1 and MATE2-K) and P-glycoprotein (P-gp) (*unpublished*). Therefore, the primary organ to eliminate HSK7653 is the kidney, and glomerular filtration is the main pathway for its excretion.

Generally, impaired renal function alters the renal excretion of drugs (Helal et al., 2012; Nolin et al., 2008), thus affecting the PK characteristics of drugs to a certain extent. Moreover, chronic kidney disease (CKD) is a common comorbidity of T2DM patients, and about 50% of T2DM patients worldwide suffer from CKD. Hence, evaluating the PK of HSK7653, a compound with a long half-life, in population with renal impairment is a necessary step in the development of new drugs and also a prerequisite for broadening the application population (Food and Drug Administration, 2020). Furthermore, elder adults account for 20% of T2DM patients, and DPP-4 inhibitors have greater potential and benefits in the treatment of elder patients with T2DM (Paolisso et al., 2012). Understanding the impact of physiology factors, such as delayed absorption (Grassi et al., 2011) and decline in renal function (Turnheim, 2004), on the systemic exposure of HSK7653 in the geriatric population is essential to preempt the development of hypoglycemia.

Currently, a physiologically-based pharmacokinetic (PBPK) model can be used to explore the influences of intrinsic and extrinsic factors on the systemic exposure of compounds through the method of *in vitro–in vivo* extrapolation. Evaluating the systemic exposure of HSK7653 in renal impairment and geriatric populations through the PBPK model is conducive to scientifically and reasonably designing the dosage regimen of clinical trials for the corresponding population. Here, we are aimed (i) to develop a mechanistic absorption and disposition model for HSK7653 based on preclinical data, such as the absorption and metabolism characteristics *in vitro*, and clinical data to accurately capture the absorption, distribution, metabolite, and excretion (ADME) characteristics of HSK7653 *in vivo* and then (ii) to assess the impact of physiological factors and food on the systemic exposure of HSK7653 so as to understand the changes in PK characteristics in specific populations, including renal impairment and elder populations, and support the dose design of clinical trial regimens.

## 2 Materials and methods

### 2.1 Overall strategy

**Fig. 2:**
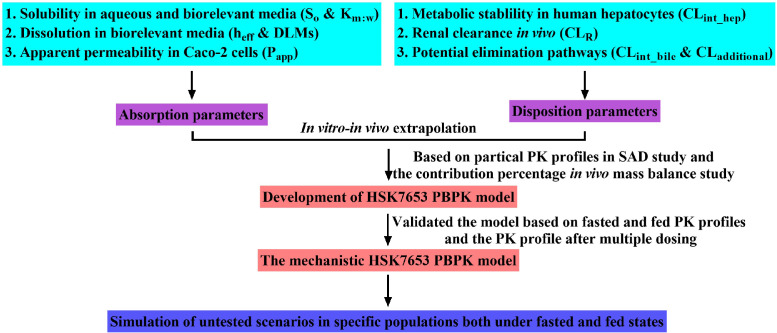
Workflow of HSK7653 PBPK model development and simulation.

The HSK7653 PBPK model was developed using an *in vitro–in vivo* extrapolation method according to the preclinical and clinical data. First, formulation-related data and absorption and metabolite characteristics were characterized *in vitro*. The model was then developed based on *in vitro* experimental data and the *in vivo* distribution characteristics of HSK7653 along with the renal clearance. The absorption model in this manuscript was developed in two ways and coupled with the same disposition model to explore more mechanistic methods to describe the absorption of HSK7653. To confirm the absorption and disposition models without simultaneous overestimation/underestimation, the model was validated by the PK study of HSK7653 IR tablets under fasted and fed states (CTR20180791) after the model was developed based on the contribution percentage of each elimination pathway *in vivo* (CTR20201559) and the PK profiles of HSK7653 in SAD study at the dose of 5 mg, 10 mg, 25 mg, 100 mg, and 150 mg (CTR20180791). Meanwhile, the model was also validated by PK data from the drug-drug interaction of HSK7653 co-administration with metformin in healthy volunteers (multiple doses, CTR20191549). Finally, the mechanistic HSK7653 PBPK model was used to evaluate the potential systemic exposure of HSK7653 IR tablets at doses of 10 mg and 25 mg in adult patients with renal impairment, elder patients with renal impairment, and geriatric populations under both fasted and fed states, so as to provide support for the dose design of the clinical trials in the development of HSK7653. Fig. 2 displays the detailed workflow of PBPK model development and simulation, and Table 1 shows the untested simulation scenarios.

**Table 1:** Simulation scenarios of HSK7653 IR tablets in specific populations.

Populations	Substrate	Single Dose (mg)
Healthy volunteers (20–60 years old)	Fasted and Fed	10 and 25
Chinese healthy volunteers (20–60 years old)	Fasted and Fed	10 and 25
Mild renal impairment population (20–60, 65–75, 75–85 and 85–95 years old)	Fasted and Fed	10 and 25
Moderate renal impairment population (20–60, 65–75, 75–85 and 85–95 years old)	Fasted and Fed	10 and 25
Severe renal impairment population (20–60, 65–75, 75–85 and 85–95 years old)	Fasted and Fed	10 and 25
Geriatric population (65–75, 75–85 and 85–95 years old)	Fasted and Fed	10 and 25

### 2.2 Equilibrium solubility of the HSK7653 active pharmaceutical ingredient (API) in various media

The equilibrium solubility of the HSK7653 API in pH 1.0 hydrochloric acid (HCl) solution, pH 4.5 acetic acid solution, pH 5.0, pH 6.5, and pH 8.0 phosphate buffer saline (PBS), FaSSGF, FaSSIF, and FeSSIF biorelevant media were investigated, respectively. Tests were performed by adding an excess amount of API to vials and stirring continuously at a speed of 200 rpm for 24 h at 37 °C. The API concentration was detected once per minute by an *in situ* fiber-optic ultraviolet (UV) dissolution real-time monitoring system (Pion Scientific Instruments, Billerica, MA, USA). All measurements were performed with *n* = 3.

### 2.3 Dissolution test of HSK7653 IR tablets in FaSSGF medium

To understand the dissolution progress of HSK7653 IR tablets in the stomach, the dissolution behavior of HSK7653 tablets (25 mg) in FaSSGF medium was evaluated *in vitro*. The calibrated USP 2 apparatus (Pion Scientific Instruments, Billerica, USA) with a paddle speed of 75 rpm was used to complete the test. At the beginning of the test, one 25 mg tablet was added into the vessel containing 300 mL of FaSSGF medium at 37 °C ± 0.5 °C. Meanwhile, the concentration of HSK7653 released into the vessel was measured by an *in situ* fiber-optic UV dissolution real-time monitoring system, and the test was conducted for 1 hour. The experiment was carried out in three groups in parallel.

### 2.4 Two-stage biorelevant dissolution test

Since the equilibrium solubility of weakly basic HSK7653 is pH dependent and bile salt dependent, it is necessary to explore whether the compound dissolved in the stomach continues to dissolve in the intestine after gastric emptying. Here, the calibrated USP 2 apparatus with a 75-rpm paddle speed was used to conduct a two-stage dissolution test. The tablets (*n* = 3, one 25 mg dosage tablet in each vessel) were first dissolved in the FaSSGF medium for 29 min. At the 30th minute, the conditioning solution (a mixture of pH 10.2 PBS and concentrated FaSSIF medium) was added rapidly into the FaSSGF medium (initial volume of 300 mL) so that the mixed solution was similar to the FaSSIF medium (terminal volume of 375 mL). The dissolution in the vessel was continued during and after the addition process. To capture the slight change in concentration after the sudden change from FaSSGF medium to the FaSSIF medium, the concentration in the vessel was detected every second over 10 min after the conditioning solution was added. Moreover, the concentration of the HSK7653 tablets released into the system at other moments was determined every minute by the Rainbow DDMS with Pion fiber optic probes (Pion Scientific Instruments, MA, USA). The test was conducted in triplicate.

### 2.5 Particle size distribution

The particle size distribution of the API that was used to prepare HSK7653 IR tablets was determined by the Malvern Mastersizer laser diffraction size analyzer (Master 2000, Malvern Instruments Limited, Malvern, UK). The determination was performed in a dry method with a Scirocco dry powder feeder, and the air was used as the dispersion agent (Tari et al., 2015). Meanwhile, the analysis results, d (0.1), d (0.5) and d (0.9) (defined as the diameter where 10%, 50% and 90% of population lies below this value), were directly output.

### 2.6 Apparent permeability of HSK7653 in Caco-2 monolayer cell lines

Intact Caco-2 monolayer cells were used to evaluate the apparent permeability of HSK7653 as previous reported (Zhang et al., 2021). HSK7653 solutions with concentrations of 1, 5, and 20 μM were added into the apical or basolateral chambers of 24-well Transwell permeable plates (polycarbonate membrane, diameter 6.5 mm, pore size 0.4 μm, Corning, NY, USA), respectively, and incubated for 2 h at 37 °C. Meanwhile, to ensure the applicability of the incubation system, propranolol (with high apparent permeability), atenolol (with low apparent permeability), and digoxin (the substrate of P-gp) were selected as positive controls. All experiments were performed in triplicate and analyzed by high-performance liquid chromatography with tandem mass spectrometry (HPLC-MS/MS). The apparent permeability coefficient (*P*_app_) of each compound under the corresponding concentration was calculated according to Eq. (1) and listed as follows:

(1) $$P_{app}=\frac{dQ}{dt}\times \frac{1}{A\times C_{0}}$$

Where *P*_app_ was the apparent permeability coefficient (cm/*s* × 10^−6^), d*Q*/d*t* (pmol/second) was the rate at which the compound appears in the receiver side, *C*_0_ (nM) was the initial concentration of the compound in the donor side, and *A* (cm^2^) represented the surface area of the cell monolayer.

### 2.7 Metabolic stability of HSK7653 in human plated hepatocytes

Since 1 μM HSK7653 solution was very stable in the 0.5 mg/mL human liver microsomes incubation system (*unpublished*), the metabolic stability of HSK7653 in primary human plated hepatocytes was investigated. Briefly, cryopreserved human hepatocytes (Bioreclamation IVT, X008001-P, 10 donors mixed, SYK) were thawed and seeded in plates followed by cell viability (90%) using trypan blue. The plated hepatocytes were then incubated with 0.05 μM HSK7653 in a humidified atmosphere incubator containing 95% air and 5% CO_2_ at 37 °C for 0, 1, 2, 4, 24, and 48 h, respectively. Reactions were stopped by removing the plates from the incubator and mixing the incubation solution with 600 μL of ice-cold stop solution (acetonitrile) at the corresponding sampling time points. Samples were centrifuged at 3220 × g for 20 min at 4 °C, and then the supernatant was collected and quantified by HPLC-MS/MS. Meanwhile, atorvastatin calcium salt and S-mephenytoin were used as the positive control group, and the experimental procedure was the same as that of HSK7653. The intrinsic clearance (CL_int_hep_) of hepatocytes to the metabolism of HSK7653 was calculated according to Eq. (2) (Lin et al., 2016), as shown below:

(2) $$CL_{int}=\ln2\times \frac{1}{t_{1\;/\;2}\left(min\right)}\times \frac{incubation\;volume\;\left(mL\right)}{10^{6}\;cells}$$

Where *CL_int_* was the intrinsic clearance of compound metabolism by hepatocytes.

### 2.8 Analysis method

The Acquity ultra performance liquid chromatography (UPLC) system (Waters, Milford, MA, USA) connected to the SCIEX API 4000 (AB Inc, Alberta, Canada) Triple quad mass spectrometer was used for the quantitative analysis. Chromatographic separation was performed on an Acquity UPLC BEH C18 column (2.1 × 50 mm, 1.7 μm; Waters). The mobile phases were (A) aqueous solution and (B) acetonitrile (HPLC grade, Fisher Chemical, Waltham, MA, USA) containing 0.1% formic acid (LC/MS grade, Fisher Scientific), respectively. Gradient elution was used as follows: 10% B at 0.01–0.10 min, 10%–95% B at 0.10–0.90 min, 95% B at 0.90–1.10 min, 95%–10% B at 1.10–1.11 min, 10% B at 1.11–1.20 min. The injection volume was 20 μL, and the flow rate of the mobile phase was maintained at 0.5 mL/min during the analysis. Mass spectrometric analysis was performed using positive electrospray ionization in multiple reaction-monitoring mode. Ion transitions were *m/z* 467.0→172.9 and *m/z* 271.1→155.3 for HSK7653 and tolbutamide (internal standard, Sigma-Aldrich/Avanti, Hamburg, Germany), respectively.

### 2.9 Development of the HSK7653 PBPK model

The HSK7653 PBPK absorption model was developed in an advanced dissolution, absorption, and metabolism (ADAM) model in a SimCYP population-based simulator (version 21, SimCYP Limited, Sheffield, UK). The ADAM model was composed of permeability parameters and formulation parameters. The average apparent permeability of HSK7653 at different incubation concentrations was used to represent the ability of HSK7653 to pass through the intestinal epithelial cell membrane. Meanwhile, the apparent permeability of the positive control group (propranolol and atenolol) in the Caco-2 incubation experiment was used as the calibration factor through the permeability calibrator embedded in the prediction toolbox of the SimCYP simulator (Ezuruike et al., 2022). Here, the dissolution profile of the HSK7653 tablets (the first method) and the diffusion layer model (DLM) (the second method) were used to describe the dissolution process of HSK7653 tablets *in vivo*, and the effects of the different methods on predicting HSK7653 absorption characteristics were compared. The dissolution profile of the HSK7653 tablets at pH 4.5 acetum (experimental methods not presented in this manuscript) was entered directly into the model to constitute the formulation parameters. The development of the DLM was complex. First, in combination with equation (3) (Pathak et al., 2017), solubility parameters including intrinsic solubility (S_o_), solubility factors (SFs), and the bile micelle:buffer partition coefficients (K_m:w_) for the ionized (K_m:w, ionized_) and un-ionized (K_m:w, un-ionized_) were estimated based on the solubility of HSK7653 in aqueous media and biorelevant media (FaSSIF and FeSSIF). The dissolution parameters, such as effective diffusion layer thickness (h_eff_) and DLM scalar (DLMs), were estimated based on equation (4) (Arora et al., 2020) by fitting the dissolution profile of HSK7653 tablets dissolved in FaSSGF medium. Due to the lag time before the HSK7653 concentration could be detected in the vessel during dissolution, disintegration parameters were also evaluated to match the dissolution profile of HSK7653 tablets in FaSSGF medium. Based on the results of the two-stage biorelevant dissolution test (see Fig. 4 in 3.2 section), the precipitation parameters in the DLM were not assessed, and the critical supersaturation ratio (CSC) and precipitation rate constant (PRC) were set to the minimum values, respectively. The particle size distribution results of the HSK7653 were input into the DLM to represent the initial value of particles in HSK7653 tablets dissolved in the stomach. The disposition model parameters of the two absorption models were the same. Using the method devised by Rodgers et al. (Rodgers et al., 2005; Rodgers and Rowland, 2006), the full PBPK model with the predicted steady state distribution volume (V_ss_) was used to describe the distribution characteristics of HSK7653 *in vivo*. Meanwhile, the tissue-to-plasma partition coefficient (K_p_) was fitted to adjust the shape of the predicted concentration-time profile and set to 4.5 in the HSK7653 PBPK model. The elimination system was composed of the intrinsic clearance of hepatocyte metabolism (CL_int_hep_), renal clearance (CL_R_), biliary clearance (CL_int_bile_), and additional systemic clearance (CL_additional_). Except for the CL_int_hep_ and CL_R_, which were obtained separately from the *in vitro* experimental results and *in vivo* clinical study results, CL_int_bile_ and CL_additional_ were obtained by fitting the contribution percentage of HSK7653 elimination through feces (7.40%) and an unknown pathway (4.10%) in the mass balance study (CTR20201559). Meanwhile, PK profiles from the SAD study at doses of 5 mg, 10 mg, 25 mg, 100 mg, and 150 mg (CTR20180791) were also used to fit and then obtain specific values for K_p_, CL_int_bile_ and CL_additional_. Subsequently, local parameter sensitivity analyses were performed separately for CL_int_bile_ and CL_additional_ with a step size of 0.005 from the same range of 0.005 to 0.05 covering the specific values (0.025 μL/min/10^6^ and 0.025 L/h, respectively) to evaluate the impact of these two pathways on model performance. Last but not least, physicochemical parameters including molecular weight (MW), protein binding ratio, blood-to-plasma partition ratio, log *P*, and pK_a_ were obtained from the experimental outcomes.

(3) $$S_{Tot}=S_{o}\times S_{oscalar}\left(t\right)\times \left(1+\frac{\left[BS\right]\left(t\right)}{C_{H_{2}O}}\times K_{m:w,\;unionised}\right)+S_{i}\left(t\right)\times \left(1+\frac{\left[BS\right]\left(t\right)}{C_{H_{2}O}}\times K_{m:w,\;ionised}\right)+S_{bound,excip}\left(t\right)$$

Where *t* was the time; *S_Tot_* was the total solubility of API in a given medium (aqueous or biorelevant medium); *S_o_* was the intrinsic solubility of the API; *S_o, scalar_* was the scalar for S_o_, which was used to capture the increased solubility of the API in a certain medium; *S_i_* was the ionized aqueous solubility; [*BS*] was the bile salt concentration in a certain biorelevant medium; C_H2O_ was the concentration of water; *K_m:w, ionized/un-ionized_* was the bile micelle:buffer partition coefficients for ionized and un-ionized species, respectively; *S_bound, excip_* was the amount of API bound to the excipient in a given excipient concentration

(4) $$DR\left(t\right)=-N\times DLM_{Scalar}\times \frac{D_{eff}\left(t\right)}{h_{eff}\left(t\right)}\times \;4\pi a\left(t\right)\times \left(a\left(t\right)+h_{eff}\left(t\right)\right)\times \left(S_{surface}\left(t\right)-C_{bulk}\left(t\right)\right)$$

Where *t* was the time; *DR.* was the dissolution rate; *N* was the number of particles; *DLM_scalar_* was the empirical scalar and the default value was 1; *D_eff_* was the effective diffusion coefficient, *a(t)* was the particle radius at time t, *h_eff_ (t)* was the effective diffusion layer thickness at time t, *S_surface_ (t)* was the concentration of drug at the particle surface at time t, and *C_bulk_ (t)* was the concentration of the drug in the bulk solution at time t.

### 2.10 Validation of HSK7653 PBPK model

The validation of the HSK7653 PBPK model was based on two clinical studies, including the SAD clinical study in Chinese healthy volunteers (CTR20180791) and the pharmacokinetic drug-drug interaction between HSK7653 and metformin in Chinese healthy volunteers after multiple dosing (CTR20191549). The trial design in SimCYP simulator was identical to the corresponding clinical trial regimen (Table S1), including demographics (age, sex, and ethnicity), dosage regimens and blood collection time points. Since there is no T2DM patient population in the population database of SimCYP simulator, all simulations were conducted with the virtual Chinese population (10 trials with 10 subjects, *n* = 100). Finally and most importantly, the predictive performance of the HSK7653 PBPK model was estimated against to two criteria: (i) the observed concentration-time profile was within the 90% confidence interval (CI) of the predicted one; (ii) the ratios of major pharmacokinetic parameters (AUC, C_max_, CL/F and V_z_/F) were within a predefined boundary of 0.5–2.0 folds.

### 2.11 PK simulation in specific populations

The potential risk of HSK7653 in patients with T2DM is hypoglycemia, which is often associated with systemic exposure. Here, we assumed that the same systemic exposure of HSK7653 would bring the same pharmacologic effects *in vivo*. The validated HSK7653 PBPK model was used to simulate untested scenarios, such as HSK7653 administration in renal impairment and geriatric populations. To precisely estimate the influence of physiological factors on HSK7653 systemic exposure, renal impairment population was divided into three subgroups according to the glomerular filtration rate (GFR). GFR at 60 to 90 min/mL/1.73 m^2^, 30 to 60 min/mL/1.73 m^2^, and 15 to 30 min/mL/1.73 m^2^ corresponded to mild, moderate, and severe renal impairment, respectively. Meanwhile, the geriatric population was also composed of three subgroups: 65–75 years old, 75–85 years old and 85–95 years old. Moreover, the elder adults with mild, moderate, and severe renal impairment were also included in the study subgroup. Because patients with renal impairment and geriatric populations included in the SimCYP simulator were developed based on the Caucasian population, the systemic exposure of HSK7653 at doses of 10 mg and 25 mg under the fasted state was simulated in “Healthy Volunteer” aged 20–60 years, and the simulated results (AUC_0-t_ and C_max_) served as the baseline to quantitatively evaluate the effects of physiological factors and food on HSK7653 systemic exposure in the specific populations. Additionally, under the assumption that the changes in physiological factors of Chinese individuals with impaired renal function and elder adults have the same influence on HSK7653 systemic exposure as the corresponding Caucasian population does, the simulation was carried out in “Chinese Healthy Volunteer” aged 20–60 years to provide some suggestions for the clinical trial regimen dose design in Chinese patient with impaired renal function and the older population. All simulations were performed under the predesigned scenarios listed in Table 1.

## 3 Results

### 3.1 Evaluation of solubility parameters based on the equilibrium solubility of HSK7653 in aqueous and biorelevant media

**Fig. 3:**
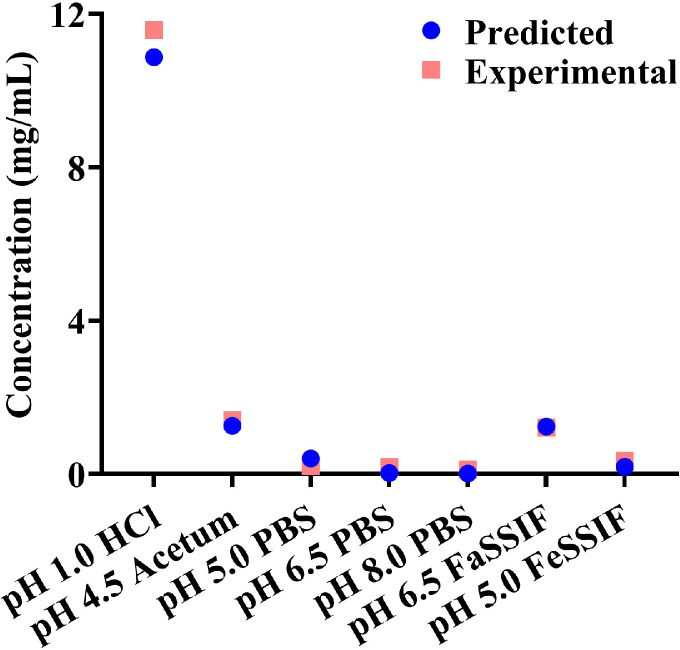
Comparison of solubility predictions (blue circles) with experimental results (pink blocks).

The equilibrium solubility of HSK7653 in the physiological pH range varied with the change in pH. Within the test pH range, the highest solubility (11.58 mg/mL) was found in the pH 1.0 HCl solution, and the lowest solubility (0.12 mg/mL) was found in pH 8.0 PBS. The solubility of HSK7653 was also dependent on bile salt. The solubility of API in FaSSIF and FeSSIF media was significantly higher than that in aqueous media at the same pH, and the solubility increased along with the increase in bile salt concentration. Furthermore, solubility parameters, representing the influence of pH and bile salts on the solubility, were estimated by Eq. (3) according to the experimental results. The predicted and experimental results are shown in Fig. 3. The predicted results were in good agreement with the experimental results, so the evaluated solubility parameters can be used as the basic parameters for the development of the absorption model by the DLM method.

### 3.2 Evaluation of dissolution parameters based on dissolution profiles in biorelevant media

**Fig. 4:**
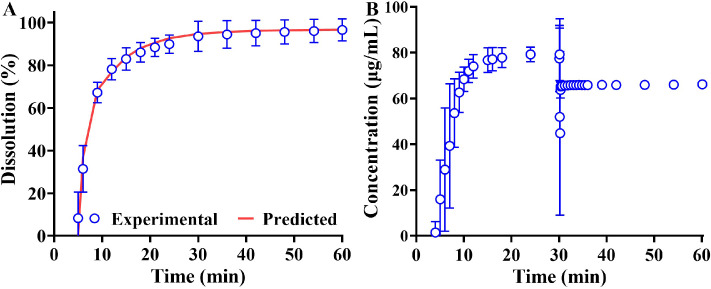
Dissolution test results of HSK7653 IR tablets (A: time-dependent dissolution percentage of HSK7653 IR tablets in 300 mL FaSSGF medium; B: dissolution profile of HSK7653 IR tablets in the two-stage dissolution test; the red line is the predicted dissolution profile, and the blue hollow circles are the experimental results).

The HSK7653 IR tablets (25 mg) were rapidly and entirely (96.6%) dissolved in the 300 mL FaSSGF medium at the paddle speed of 75 rpm within 1 hour (Fig. 4A). While, there was a lag time (about 4 min) before detecting the concentration of HSK7653 in the vessel, which might be caused by the instantaneous disintegration limit. Moreover, there was a gradient change in the concentration of HSK7653 in the two-stage dissolution test, but no precipitation occurred after the medium changed from FaSSGF to FaSSIF. The solubility of API in FaSSGF and FaSSIF medium was about 5.31 mg/mL and 0.342 mg/mL, respectively, so HSK7653 tablets (25 mg) could be completely dissolved in the 300 mL FaSSGF and 375 mL FaSSIF medium, respectively. Therefore, the concentration variation in the two-stage dissolution test (Fig. 4B) was due to the volume difference after addition of the conditioning solution. Accordingly, it was unnecessary to evaluate the precipitation parameters for the development of DLM, which (CSC and PCR) were set to minimum values in DLM. Most importantly, the dissolution parameters, such as h_eff_ and DLMs, were estimated by Eq. (4) from the dissolution profile in 300 mL FaSSGF medium. Meanwhile, the disintegration lag time of the formulation was evaluated with the method of Weibull function, so that the dissolution profile could be captured by Eq. (4). In Fig. 4A, the fitted profile matched the experimental results well and the evaluated parameters were used as the parameters of the DLM absorption model.

### 3.3 Particle size distribution

Since particle size is a crucial parameter for the development of absorption model by DLM method (HIGUCHI and HIESTAND, 1979), the particle size distribution of HSK7653 API was measured by the Malvern Mastersizer laser diffraction analyzer. The d (0.1), d (0.5) and d (0.9) of API were 1.313, 3.288 and 8.163 μm, respectively. And all these values were input directly into the model as elements of HSK7653 IR tablets dissolution evaluation in the model.

### 3.4 Apparent permeability of HSK7653

The apparent permeability (*P*_app A→B_) of HSK7653 was 25.3 × 10^−6^, 29.3 × 10^−6^ and 40.3 × 10^−6^ cm/s at concentrations of 1, 5, and 20 μM in the Caco-2 cell lines, respectively, indicating that HSK7653 is a high-permeability compound. Meanwhile, the efflux ratio (the ratio of *P*_app A→B_ to *P*_app B→A_) of HSK7653 was 1.2, 1.2, and 1.0 at concentrations of 1, 5, and 20 μM, respectively, suggesting that HSK7653 is not a specific substrate of P-gp. Considering the high solubility of HSK7653 *in vitro*, the average value of HSK7653 *P*_app_ at different incubation concentrations was used to represent the apparent permeability in the absorption model to narrow down the potential effect of experimental conditions, especially the incubation concentration, on the permeability. Moreover, the *P*_app_ values of propranolol, atenolol, and digoxin were 33.0 × 10^−6^, 0.257 × 10^−6^ and 1.85 × 10^−6^ cm/s, respectively, indicating that the incubation system was robust. These results (propranolol and atenolol) were used as calibration parameters for the apparent permeability of HSK7653 in the absorption model.

### 3.5 Metabolic stability of HSK7653 in human plated hepatocytes

Because HSK7653 is very stable in human liver microsomes (*unpublished*), it is quite imperative to evaluate the metabolic stability of HSK7653 in human hepatocytes. The half-lives (t_1/2_) of HSK7653, atorvastatin calcium salt, and S-mephenytoin (positive control groups) in the human hepatocytes were 293.5, 14.4, and 21.7 h, respectively, which suggests that HSK7653 is metabolized slowly in the human hepatocyte incubation system. Considering that about 15.37% of the parent drugs were eliminated by metabolism (mass balance study, *unpublished*), we input the CL_int_hep_ (0.056 μL/min/10^6^) based on the *in vitro* experiments into the disposition model to extrapolate the metabolic characteristics of HSK7653 *in vivo*.

### 3.6 Development and validation of the HSK7653 PBPK model

**Fig. 5:**
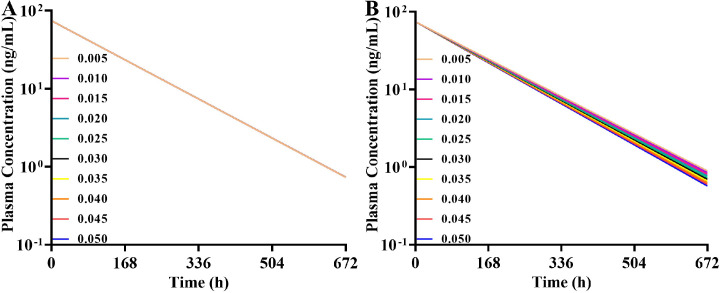
Parameter sensitivity analysis results (A: sensitivity analysis for CL_int_bile_; B: sensitivity analysis for CL_additional_).

**Fig. 6:**
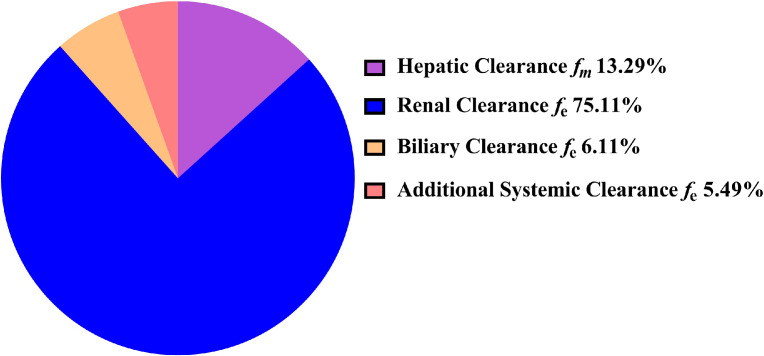
Predicted contribution percentage of each pathway to HSK7653 elimination *in vivo*.

In this manuscript, we constructed the ADAM absorption model using the dissolution profile method and DLM method, respectively. The same disposition model was severally coupled with these two different absorption models to capture the absorption and disposition characteristics of HSK7653 *in vivo*. Table 2 summarizes the parameters of these two different models. Here, we collected the CL_int_hep_ and CL_R_ from *in vitro* experimental results and *in vivo* clinical trial data without further optimization. Meanwhile, we assumed that the unchanged drugs in feces (7.40%) and unrecovered radioactive substances (4.10%) in the mass balance study were eliminated by bile and additional system clearance pathways, respectively. The CL_int_bile_ and CL_additional_ were obtained by fitting the contribution percentage of HSK7653 in the mass balance study (CTR20201559) and the PK profiles in the SAD studies at doses of 5 mg, 10 mg, 25 mg, 100 mg, and 150 mg (CTR20180791). The effects of these two elimination pathways on HSK7653 exposure were estimated by parameter sensitivity analysis and the results are shown in Fig. 5. Within the estimated range of 0.005 to 0.05, biliary excretion had little effect on the systemic exposure of HSK7653 (Fig. 5A). In contrast, the systemic exposure of HSK7653 decreased with increasing CL_additional_ (Fig. 5B). Fig. 6 presents the predicted contribution percentage of each organ to the elimination of HSK7653 at a dose of 25 mg. The predicted values were almost identical to the *in vivo* results, and the predicted results of the HSK7653 PBPK model developed by the two methods were consistent. Therefore, the disposition model of HSK7653 could accurately capture the elimination characteristics of HSK7653 *in vivo*. Because the PK characteristics of 50 mg HSK7653 under fasted and fed states were studied in the SAD clinical trial (CTR20180791), models were first validated by the PK data collected from these two clinical studies. Both absorption models could well capture the absorption characteristics of HSK7653 under fasted and fed states, and the ratios of the predicted AUC, C_max_ and CL/F to the observed ones were within the range of 0.80- to 1.25-fold, indicating that the absorption models developed by the two different methods had a similar ability to capture the absorption characteristics of HSK7653 *in vivo*. On the basis of these validated results, the HSK7653 PBPK models were validated through absorption and disposition mechanisms, which showed that the absorption and disposition characteristics of the HSK7653 PBPK model were reasonably balanced. Furthermore, the models were also validated with PK data from a multiple dose clinical study (CTR20191549). The predictive performance of these two models met the predefined standards, including that the observed concentration-time points were within the 90% CI of the predicted points (Figure S1 and Figure S2 in the supplemental file), and the main PK parameter ratios (AUC, C_max_, CL/F, and V_z_/F) were within the range of 0.5- to 2.0-fold; in particular, the ratios of most PK parameters were within the range of 0.80- to 1.25-fold (Fig. 8, Table S2, and Table S3). Accordingly, the mechanism driven HSK7653 PBPK model can be used to simulate untested scenarios in specific populations.

**Table 2:** Summary of the HSK7653 PBPK model parameters by two development methods.

Parameter	Input Value	Source
Physicochemical properties		
Molecular weight (g/mol)	466.43	
Log *P*	1.42	Measured (*in vitro*)
Compound type	Monoprotic base	
*pK*_a_	6.75	Measured (*in vitro*)
Blood-to-plasma partition ratio	0.806	Measured (*in vivo*)
Fraction unbound in plasma	0.143	Measured (*in vivo*)
Absorption		
Absorption model	ADAM	
Permeability Assay	Caco-2	
Apical pH: Basolateral pH	7.4: 7.4	
Activity		
Caco-2(10^−^6 cm/s) (HSK7653)	31.633	Measured (*in vitro*)
Caco-2(10^−^6 cm/s) (Propranolol)	33.0	Measured (*in vitro*)
Caco-2(10^−^6 cm/s) (Atenolol)	0.257	Measured (*in vitro*)
DLM Particle Handling Model	Particle Population Balance	
Formulation (The first method)	Immediate Release	
Dissolution Profile (All)		
Fasted Time (h) 1	0.00	
Fasted Dissolution (%) 1	0.00	Measured (*in vitro*)
Fasted CV (%) 1	0.00	Measured (*in vitro*)
Fasted Time (h) 2	0.167	
Fasted Dissolution (%) 2	70.52	Measured (*in vitro*)
Fasted CV (%) 2	9.31	Measured (*in vitro*)
Fasted Time (h) 3	0.333	
Fasted Dissolution (%) 3	95.97	Measured (*in vitro*)
Fasted CV (%) 3	2.12	Measured (*in vitro*)
Fasted Time (h) 4	0.50	
Fasted Dissolution (%) 4	100.0	Measured (*in vitro*)
Fasted CV (%) 4	1.30	Measured (*in vitro*)
Formulation (The second method)	Immediate release: DLM Model	
Solid State Specific Parameters	Solid state 1	
Dissolution Type	Solubility	
Solubility Type	Intrinsic (User)	
Solubility (mg/mL)	0.007	Predicted by SIVA (based on *in vitro* data)
Salt Limited Solubility Model	Solubility Factors	
Solubility Factors 1	1553	Predicted by SIVA (based on *in vitro* data)
Supersaturation Precipitation Model	First order	
Precipitation Model	Model 1	
PRC (Precipitation Rate Constant)	Global	
PRC (1/h)	1.00E-04	Minimum Value
CSR (Critical Supersaturation Ratio)	Global	
CSR value	1.000	Minimum Value
Particle size distribution	Log Normal	
Dispersion Type	Polydispersed	
d (0.1) (μm)	1.313	Measured (*in vitro*)
d (0.5) (μm)	3.288	Measured (*in vitro*)
d (0.9) (μm)	8.163	Measured (*in vitro*)
Particle density (g/mL)	1.2	Default
DLM Scalar	All Segments	
DLM Scalar values	3.009	Predicted by SIVA (based on *in vitro* data)
h_eff_ method selected	Hintz-Johnson	
h_eff_ cut-off value (μm)	36.345	Predicted by SIVA (based on *in vitro* data)
Log *K*_m:w, neutral_	5.645	Predicted by SIVA (based on *in vitro* data)
Log *K*_m:w, ion_	1.00E-06	Predicted by SIVA (based on *in vitro* data)
Distribution		
Distribution model	Full PBPK model	
*V*_ss_ input type	Predicted	
Prediction Method	Method 2	
Tissue: Plasma Partition Coefficients	Predicted	
*K*_p_ scalar	4.5	Fitted (based on *in vivo* data)
Elimination		
Clearance type	Enzyme kinetics	
Additional Hep CL_int_ (μL/min/10^6^)	0.056	Measured (*in vitro*)
Biliary CL_int_ (μL/min/10^6^)	0.025	Fitted (based on *in vivo* data)
CL_R_ (L/h)	0.373	Measured (*in vivo*)
Additional Systemic Clearance (L/h)	0.025	Fitted (based on *in vivo* data)

**Fig. 7:**
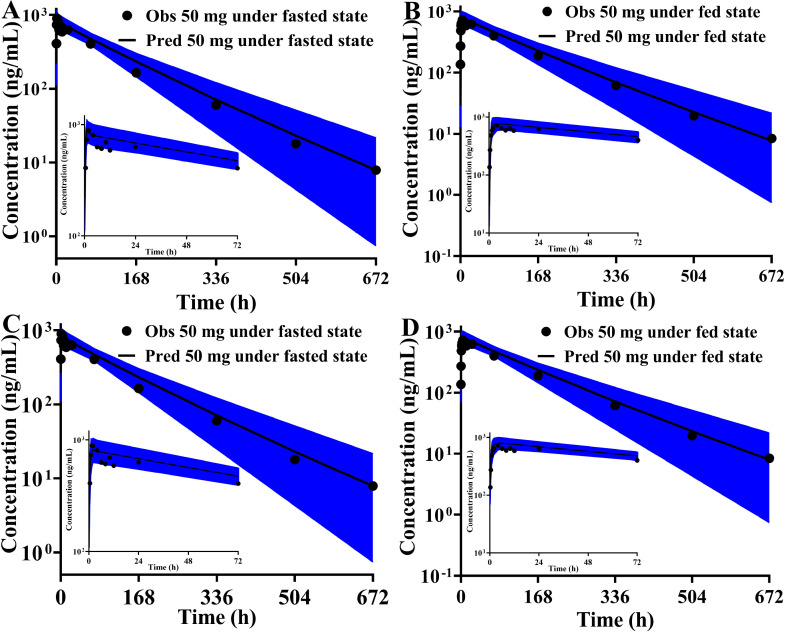
Validation results of HSK7653 at a dose of 50 mg under fasted (A and C) and fed states (B and D). (The predicted results in Fig. 7A and B are based on the model developed by the first method, and the predicted results in Fig. 7C and D are based on the model developed by the second method; the black line is the mean of the predicted results, the black circles are the observed values, and the blue color range is the 90% CI of the predicted results).

**Fig. 8:**
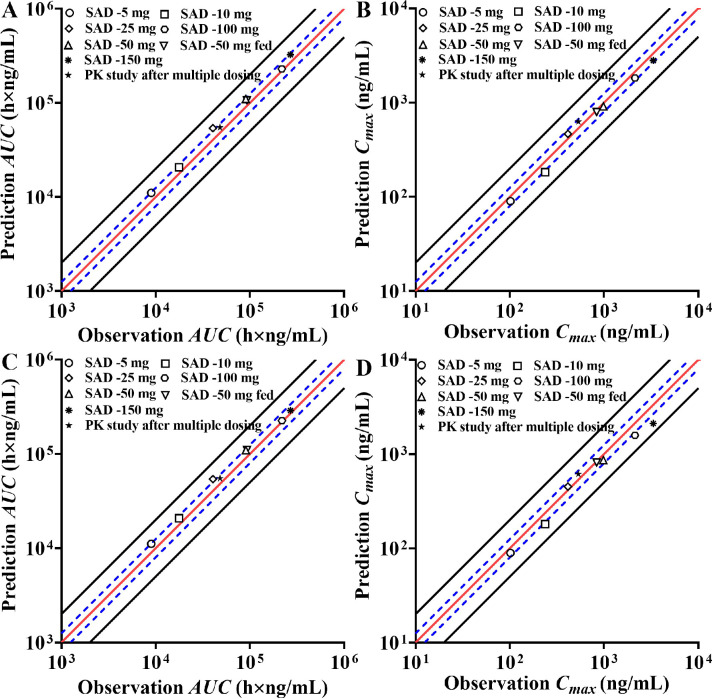
Comparison results of predicted AUC (A and C) and C_max_ (B and D) over the observed values. (The predicted results in A and B are based on the model developed by the first method, and the predicted results in C and D are based on the model developed by the second method).

### 3.7 Simulation of untested scenarios

**Fig. 9:**
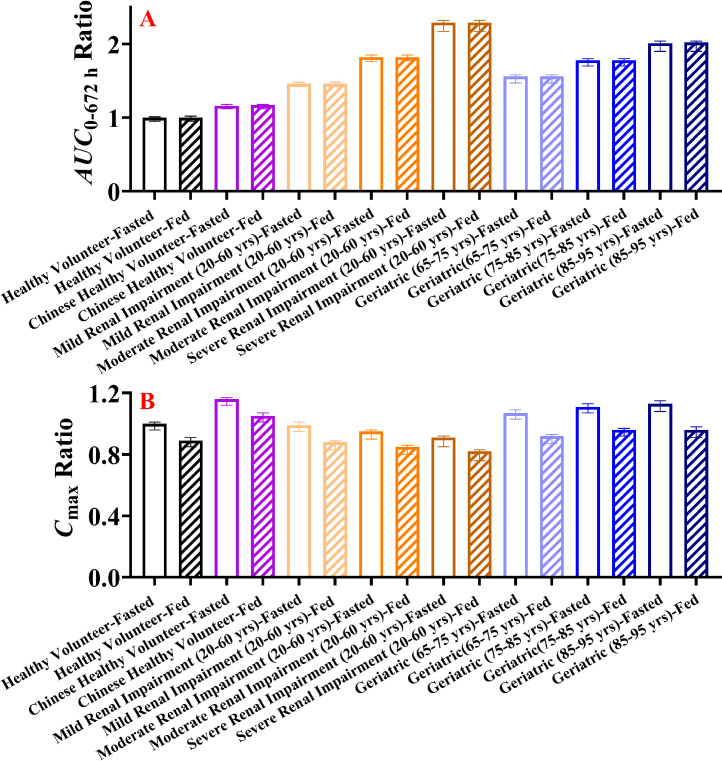
Predicted relative changes of HSK7653 systemic exposure in adult patients with renal impairment and the geriatric population compared with healthy volunteers. (A: changes in AUC_0–_672 h; B: changes in C_max_; Note: The data present in Fig. 9 are from the simulation results of HSK7653 at the dose of 10 mg with the PBPK model developed by the second method).

**Fig. 10:**
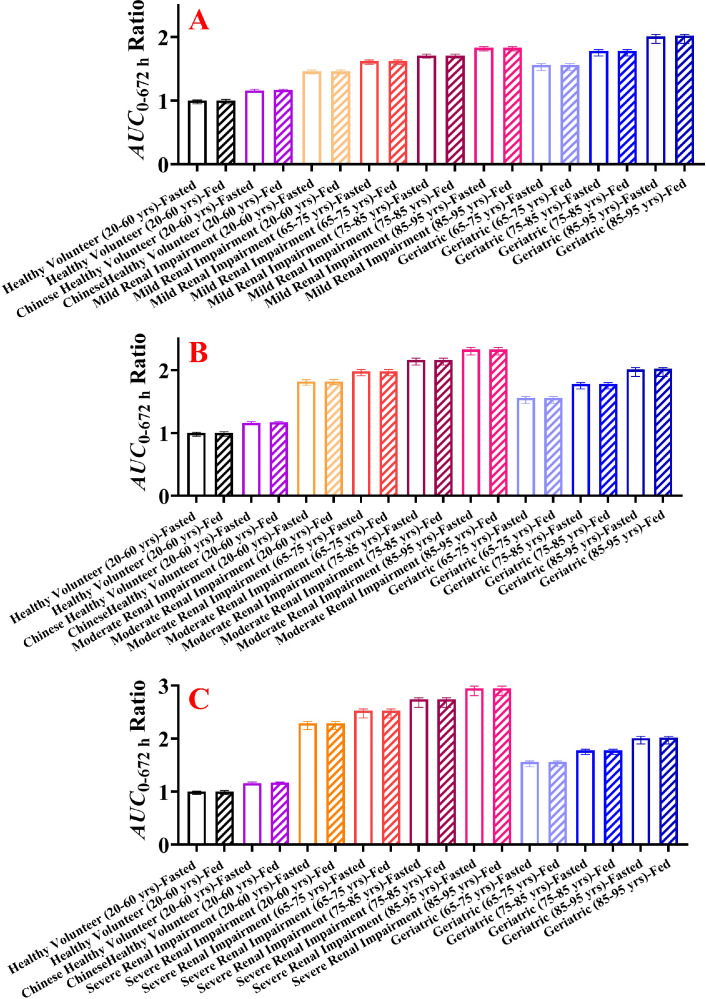
Comparison of effects of renal impairment and aging on HSK7653 systemic exposure (A: elder patients with mild renal impairment; B: elder patients with moderate renal impairment; C: elder patients with severe renal impairment; Note: The data present in Fig. 10 are from the simulation results of HSK7653 at a dose of 10 mg with the PBPK model developed by the second method).

The systemic exposure of HSK7653 at doses of 10 mg and 25 mg under fasted and fed states was simulated in “Healthy Volunteer” and “Chinese Healthy Volunteer” aged 20–60 years; in mild, moderate, and severe adult/elder populations with renal impairment; and in geriatric populations of different age groups. Fig. 9, Fig. 10 display the predicted results of the relative changes in HSK7653 systemic exposure. The relative changes in systemic exposure of HSK7653 at 10 mg and 25 mg were the same, and there was no significant difference between the predicted results of the two PBPK models using different model development methods. In this manuscript, we present only the ratios of AUC_0–672_ _h_ and*/*or C_max_ predicted by the second method at a dose of 10 mg in specific populations to that of healthy volunteers (20–60 years old). Compared with the baseline, the systemic exposure (AUC) of HSK7653 increased by 46%, 82%, and 129%, respectively, in mild, moderate, and severe adult renal impairment population under the fasted state. Meanwhile, systemic exposure increased by 56%, 78%, and 101% in the 65–75, 75–85 and 85–95 age groups, respectively. Moreover, elder patients with mild, moderate, and severe renal impairment had significantly increased systemic exposure, and the changes in the AUC were within the range of 62%–83%, 98%–133% and 153%–195%, respectively. Furthermore, food had no effect on systemic exposure (AUC) in the same simulated population, which was consistent with the 3% increase in AUC under the fed state compared with that under the fasted state in the clinical study. Additionally, the C_max_ of HSK7653 in patients with renal impairment decreased by 5%–9% and 15%–18% under the fasted and fed states, respectively. In elder patients, the C_max_ of HSK7653 increased by 7%–13% under the fasted state and decreased by 4%–8% under the fed state. Last but the most important, the systemic exposure of HSK7653 in Chinese healthy volunteers was higher (16%) than that in healthy volunteers under the fasted state, indicating that there was no significant ethnic difference between the Chinese and Caucasian populations. Therefore, the predicted results have been used as a reference for the clinical trial dose design in corresponding specific populations (CTR20221952).

## 4 Discussion

HSK7653 is a weakly basic and highly permeable compound with high solubility in pH 1.0 HCl solution and low solubility in the weakly acidic/basic buffer solutions. Meanwhile, bile salts can dramatically improve the solubility of HSK7653 at the same pH conditions. The metabolism of HSK7653 is very stable in human liver microsomes (*unpublished*) and relatively stable in human hepatocytes. It is difficult to evaluate the ADME characteristics based on the preclinical data, but the PBPK model can serve as a bridge for constructing *in vitro* and *in vivo* relationships by extrapolation and can be used to estimate potential systemic exposure in specific populations. In this manuscript, we used two methods to develop the absorption model. The first method is to directly calculate the dissolution rate of IR formulations *in vivo* according to the *in vitro* dissolution profile (Ezuruike et al., 2022). Generally, the pH of the fasted stomach was between 1 and 2 (Efentakis and Dressman, 1998), so we used the dissolution profile of the HSK7653 tablets in a pH 2.0 HCl solution to establish the absorption model first. However, the absorption phase of HSK7653 was underestimated. Then, we used the dissolution profile of HSK7653 tablets in pH 4.5 acetum to develop the absorption model, which improved the ability to predict the systemic exposure of HSK7653. Considering that the success of the model prediction depends entirely on the dissolution profile and that the selection of the dissolution profile has certain randomness and blindness, the absorption model developed by the DLM was the second method used to develop HSK7653 PBPK model in this manuscript.

DLM has many advantages, such as the evaluation of the solubilization mediated by bile micelles and simulation of the precipitate phenomenon produced in the intestine after the gastric emptying of dissolved compounds in the stomach (Arora et al., 2020; Pathak et al., 2017). Therefore, DLM has a high success rate in predicting complex absorption mechanisms, including the effect of food on the absorption of weakly basic insoluble compounds. Here, the HSK7653 PBPK absorption model was also developed by DLM. Since the DLM dissolution model calculates the potential dissolution rate of the IR formulation *in vivo* based on the *in vitro* solubility and dissolution-related parameters and then simulates the dissolution process of IR formulations *in vivo* (Arora et al., 2020), we first carried out the solubility tests of HSK7653 API in aqueous buffer solutions/biorelevant media and the dissolution tests of HSK7653 formulation in FaSSGF medium/two-stage medium. The crucial parameters affecting the dissolution rate were estimated by the Wang and Flanagan methodology (Wang and Flanagan, 1999) according to the experimental data, which provided a mechanistic approach for the development of an absorption model. Moreover, to avoid overparameterization, the precipitation rate constants in the DLM were set to the minimum value, rather than using the default values in the model, meaning that almost no precipitation was generated during gastric emptying. Accordingly, without further optimization of the absorption model developed by the DLM method, the absorption phase *in vivo* can be reasonably captured under both fasted and fed states. Although the predictive performance of the HSK7653 PBPK model developed by the two methods is similar, the second method may more mechanistically describe the absorption characteristics of HSK7653 in specific populations*,* especially in the elder population, who has physiological characteristics of delayed absorption. Meanwhile, it may be more reliable to understand the potential systemic exposure of HSK7653 in specific populations under the fed state by the second method, even though food effect studies have been conducted in healthy volunteers aged 21–47 years.

Although HSK7653 is relatively stable in human hepatocytes with a very small intrinsic clearance (0.056 μL/min/10^6^), the role of liver metabolism in the elimination of HSK7653 *in vivo* cannot be ignored. Based on the *in vitro–in vivo* extrapolation method, the intrinsic clearance could indeed describe the metabolic pathway of HSK7653 *in vivo,* and the contribution percentage in the model was 13.3%, which was close to the observed value (about 15.4%). In fact, there are unextracted radioactive substrates in urine (1.99%) and feces (2.99%) that can be detected by UPLC/Q-TOF MS (*unpublished*). Therefore, hepatic metabolism may be underestimated by approximately 4.98%. Moreover, we included unconfirmed elimination pathways in the model by assuming that biliary clearance accounted for the unchanged HSK7653 in the feces and that additional clearance accounted for the percentage of radioactivity not recovered (theoretical value, 100%, minus the detected value, 95.9%, *unpublished*). The bioavailability of HSK7653 in rats (94.5%, *unpublished*) and in beagle dogs (70.9%, *unpublished*) may not support this assumption because orally administered HSK7653 may not be absorbed and may be directly excreted in the feces. However, the predicted bioavailability (98.9%) of HSK7653 in humans based on the HSK7653 PBPK model is not affected by whether the model includes the biliary excretion pathway, as biliary excretion has little effect on the systemic exposure of HSK7653 (sensitivity analysis result). Instead, it affects the contribution percentage of other elimination pathways. Meanwhile, a biliary drainage study in rats showed that approximately 16.6% of the radioactivity was recovered (*unpublished*), and approximately 62.1% of the radioactivity was recovered in feces after oral administration of HSK7653 to rats (*unpublished*), indicating that biliary excretion is an important route for the elimination of HSK7653 and/or its metabolites from rats. Considering the significant differences between species, the unchanged HSK7653 in feces (7.40%) was considered as the contribution of biliary excretion. The predicted contribution percentage of renal clearance (75.1%) was higher than the observed percentage (68.1%), even though the percentage of unrecovered radioactivity (4.1%) may be excreted unchanged in the urine beyond the urine recovery time after dosing. Although the contribution percentage of renal clearance in the model can be reduced by increasing the clearance values for biliary excretion and additional systemic clearance in the model (the values obtained by fitting), the contribution percentage of hepatic metabolism in the model will be reduced at the same time. This is because the renal clearance was derived from *in vivo* clinical studies and the intrinsic clearance of hepatic metabolism was obtained from *in vitro* experiments. Similarly, as the contribution percentage of hepatic metabolism increases, so does the contribution percentage of renal clearance. The disposition model is reasonable given the individual differences in the results of six sample sizes in the mass balance study.

HSK7653 is a potentially potent oral DPP-4 inhibitor for patients with T2DM. In this manuscript, the validated results of the HSK7653 PBPK model are presented only for those validated by PK data from healthy volunteers. Indeed, population pharmacokinetic modeling has shown no statistical differences in PK characteristics between healthy volunteers and T2DM patients (*unpublished*). In addition, we used PK data from T2DM patients (CTR20182505, *unpublished*) to validate the HSK7653 PBPK model, and the validated results met the predefined criteria in “Section 2.10”. Therefore, the simulation results in healthy volunteers based on the HSK7653 PBPK model can be extrapolated to T2DM patients. Considering that the PK studies in T2DM patients (CTR20182505) have not been published, we did not present the validated results in this manuscript. Although we assumed that changes in physiological factors in Chinese individuals with impaired renal function and the elderly would have the same influence on the systemic exposure of HSK7653 as in the corresponding Caucasian population, this method was validated by another drug's PBPK model (Zhang et al., 2023), whose predicted changes in the Caucasian specific populations agreed well with the observed changes in the Chinese elderly and hepatic impairment population (Zhang et al., 2023). Meanwhile, we also evaluated the systemic exposure of HSK7653 in Chinese elderly and Caucasian elderly using SimCYP software version 22, which includes Chinese geriatric population. The systemic exposure (AUC) in Chinese aged 65–75, 75–85, and 85–95 years increased by 51%, 73%, and 96%, respectively, and in Caucasians aged 65–75, 75–85, and 85–95 years increased by 56%, 78%, and 100%, respectively. Thus, this result also suggests that changes in physiological factors such as organ function or body composition in the elderly can be similarly modeled in predicting drug exposure, regardless of ethnicity. Accordingly, it may be reasonable to assume that the changes in physiological factors are similar in these two populations with impaired renal impairment.

Generally, physiological senility and CKD-induced decline in organ function can reduce the elimination of HSK7653, thus increasing systemic exposure *in vivo* (Bailie et al., 2005; Boss and Seegmiller, 1981), which may lead to adverse events. Therefore, we evaluated the effects of physiological factors on the HSK7653 systemic exposure in patients with renal impairment and geriatric populations under fasted and fed states. It is worth noting that the C_max_ of HSK7653 decreased in the renal impairment populations compared to that in healthy volunteers, which was supposed to be increased. In fact, C_max_ is more related to the absorption and distribution processes. Compared to the virtual population of healthy volunteers in SimCYP, the mean gastric residence time was increased in the moderate and severe renal impairment populations, and the mean fluid and dissolved drug residence times were decreased in the mild, moderate, and severe renal impairment populations. Therefore, changes in physiological parameters may be responsible for the predicted decrease in C_max_. Under the assumption that the same systemic exposure of HSK7653 will produce the same pharmacologic effect *in vivo*, the exposure of HSK7653 in specific populations can be used as an indicator to help researchers make decisions regarding dose in the design of clinical trial regimens. Moreover, there is a relatively flat exposure-response relationship between HSK7653 systemic exposure (within a certain AUC and C_max_ range) and glycosylated hemoglobin (*unpublished*), providing additional supportive information for the dose decisions. Because the predicted systemic exposure of HSK7653 in specific populations at a dose of 10 mg (clinically recommended dose) was within the range of the exposure-response analysis results, 10 mg was used as the clinical trial dose in the population with renal impairment (CTR20221952) according to the dose-exposure and exposure-effect relationships. Additionally, food could reduce the value of C_max_ and delay the time to reach the peak concentration (T_max_), but it had no effect on the AUC. Considering that the predicted C_max_ of HSK7653 in specific populations under the fed state is still within the range of the relatively flat exposure-response analysis results and the elimination half-life of HSK7653 is long, slight changes in T_max_ and C_max_ may not affect the onset time and efficacy of HSK7653 in the clinical study under the fed state *in vivo*.

Among patients with T2DM, 50% suffer from CKD, which is also common in the elder adults (Thomas et al., 2016). CKD is clinically defined as increased urinary albumin excretion and/or decreased GFR (Bailie et al., 2005). Because the population with renal impairment in SimCYP was developed and classified based on decreased GFR, the effect of increased urinary albumin excretion on HSK7653 systemic exposure in renal impairment and elder populations cannot be estimated by the PBPK model in this manuscript. DPP-4 is expressed in human kidneys and up-regulated in the glomeruli of patients with diabetic nephropathy (Sharkovska et al., 2014). Meanwhile, DPP-4 inhibitors are sometimes able to reduce albuminuria in T2DM patients with renal dysfunction (Groop et al., 2013; Udell et al., 2015) and attenuated kidney injury in induced diabetic rats (Liu et al., 2012; Sharkovska et al., 2014). The pharmacodynamic differences of HSK7653 must also be further explored in clinical studies, even though the exposure-response is relatively flat in a certain range of AUC/C_max_ (*unpublished*). Moreover, the complex disease mechanisms of diabetes are still not included in the SimCYP population database. Therefore, the predicted results of HSK7653 in specific populations in this manuscript need to be validated by clinical practice.

## 5 Conclusions

The mechanism the HSK7653 PBPK model was developed to simulate the absorption and disposition characteristics of HSK7653 IR tablets *in vivo*. Combined with the specific populations in SimCYP, including adult patients with renal impairment, elder patients with renal impairment and the geriatric population, the influences of physiological factors and food on the systemic exposure of HSK7653 were estimated. Based on the HSK7653 systemic exposure in healthy volunteers aged 20–60 years under a fasted state, changes in HSK7653 systemic exposure in specific populations were obtained. The predicted systemic exposure (AUC) of HSK7653 increased by 46%–129% in adult patients with renal impairment population, 56%–101% in people aged 65–95 years, and 62%–195% in the elder patients with mild, moderate, and severe renal impairment. Moreover, food had no impact on systemic exposure. Considering that HSK7653 systemic exposure in different untested scenarios at a dose of 10 mg is within the relatively flat exposure-response range, HSK7653 can be first administered at a dose of 10 mg in the clinical trial with specific populations.

## CRediT authorship contribution statement

**Miao Zhang:** Data curation, Formal analysis, Investigation, Methodology, Project administration, Visualization, Writing – original draft. **Shudong Zhang:** Investigation, Methodology. **Zhiheng Yu:** Investigation, Methodology, Validation. **Xueting Yao:** Investigation, Methodology, Project administration. **Zihan Lei:** Validation. **Pangke Yan:** Project administration, Resources, Writing – review & editing. **Nan Wu:** Project administration, Resources, Writing – review & editing. **Xu Wang:** Project administration, Resources, Writing – review & editing. **Qin Hu:** Methodology. **Dongyang Liu:** Conceptualization, Funding acquisition, Investigation, Methodology, Project administration, Resources, Software, Supervision, Writing – review & editing.

## Declaration of Competing Interest

The authors declare that they have no known competing financial interests or personal relationships that could have influenced the work reported in this paper.

## Data Availability

Data is contained within the article.

## Appendix Supplementary materials

Image, application 1
