# Supplementary material for: Dose decision of HSK7653 oral immediate release tablets in specific populations clinical trials based on mechanistic physiologically-based pharmacokinetic model
Source: Eur J Pharm Sci. 2023 Oct 1;189:106553. doi: 10.1016/j.ejps.2023.106553 (PMC10485820; doi:10.1016/j.ejps.2023.106553)
Supplement: Supplementary file 1 [file mmc1.docx]

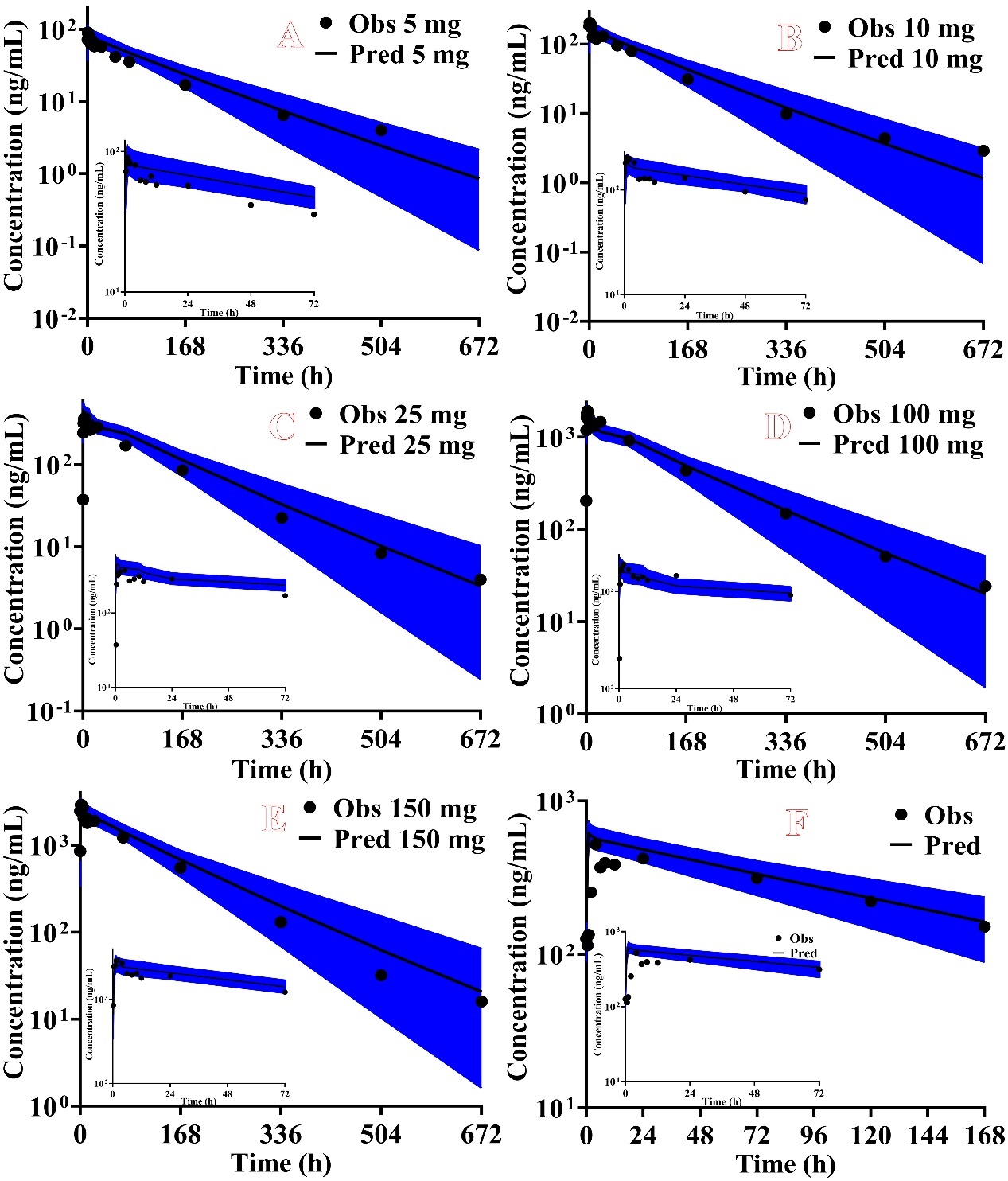


**Figure S1** Validated results of the HSK7653 PBPK model developed by the first method. (A, B, C, D and E were validated by the PK data from SAD clinical study, and F was validated by the PK data from HSK7653 coadministration with metformin in healthy volunteer following multiple dosing; the black spots are the observed values, the black line is the predicted concentration-time profile, and the blue area is the 90% CI of the predicted results)


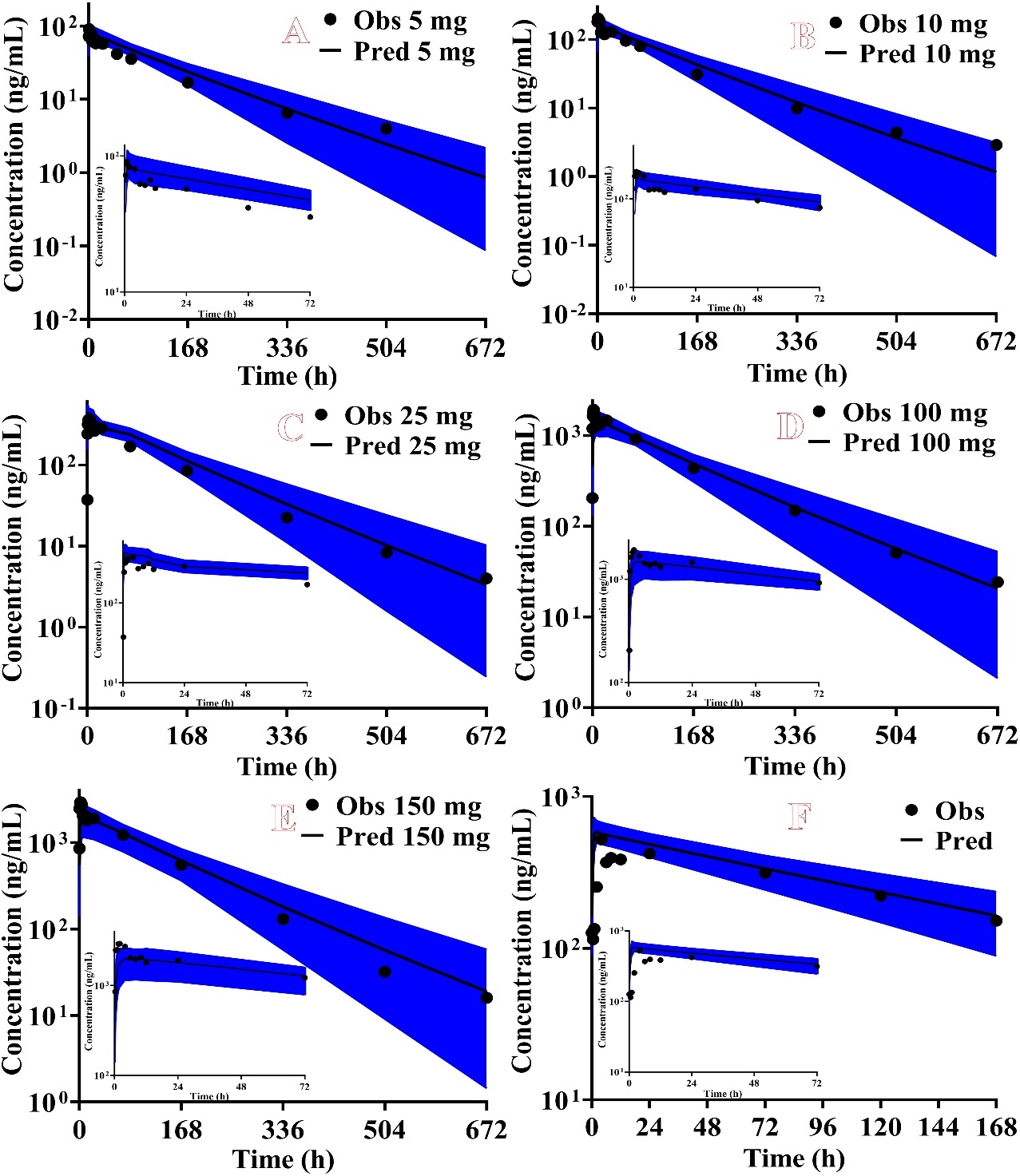


**Figure S2** Validated results of the HSK7653 PBPK model developed by the second method. (A, B, C, D and E were validated by the PK data from SAD clinical study, and F was validated by the PK data from HSK7653 coadministration with metformin in healthy volunteer following multiple dosing; the black spots are the observed values, the black line is the predicted concentration-time profile, and the blue area is the 90% CI of the predicted results)

**Table S1** Summary of the detailed trial design about HSK7653 PBPK model validation

| No. | Dosage | Subject | Age (years) | Fasted/  Fed | Male/  Female | BMI  (kg/m^2^) | Body weight  (kg) | Sample size | Use for model development | Sampling time |
| --- | --- | --- | --- | --- | --- | --- | --- | --- | --- | --- |
| Figure S1/2 A | Single dose: 5 (mg)  (CTR20180791) | Chinese healthy volunteers | 21-47 | Fasted | 4/2 | 22.6 | 61.1 | 6 | Training | 0, 0.5, 1, 1.5, 2, 4, 6, 8, 10, 12, 24, 48, 72, 168, 336, 504, 672 h |
| Figure S1/2 B | Single dose: 10 (mg)  (CTR20180791) |  | 18-32 | Fasted | 3/3 | 22.2 | 62.6 | 6 | Training |  |
| Figure S1/2 C | Single dose: 25 (mg)  (CTR20180791) |  | 20-48 | Fasted | 3/3 | 20.3 | 58.5 | 6 | Training | 0, 0.25, 0.5, 1, 1.5, 2, 4, 6, 8, 10, 12, 24, 72, 168, 336, 504, 672 h |
| Figure 6 A/C | Single dose: 50 (mg)  (CTR20180791) |  | 21-47 | Fasted | 7/5 | 23.4 | 65.0 | 12 | Testing |  |
| Figure 6 B/D | Single dose: 50 (mg)  (CTR20180791) |  | 21-47 | Fed | 7/5 | 23.4 | 65.0 | 12 | Testing |  |
| Figure S1/2 D | Single dose: 100 (mg)  (CTR20180791) |  | 32-45 | Fasted | 4/2 | 22.8 | 63.2 | 6 | Training |  |
| Figure S1/2 E | Single dose:150 (mg)  (CTR20180791) |  | 22-49 | Fasted | 6/6 | 23.8 | 63.9 | 12 | Training |  |
| Figure S1/2 F | Multiple dose:  D1: 35 (mg); D8/D15/D22 (25 mg) (CTR20191549) |  | 23-48 | Fasted | 11/11 | 24.2 | 64.9 | 22 | Testing | D22: 0.5, 1, 2, 4, 6, 8, 12, 24, 72, 120, 168 h |

Table S2 Comparison of the predicted and observed PK parameters based on the first method

| Dose  (mg) | AUC  (ng/mL×h) | | | C_max_  (ng/mL) | | | CL  (L/h) | | | V_Z_/F  (L/kg) | | |
| --- | --- | --- | --- | --- | --- | --- | --- | --- | --- | --- | --- | --- |
|  | Pred | Obs | Ratio | Pred | Obs | Ratio | Pred | Obs | Ratio | Pred | Obs | Ratio |
| 5-fasted | 11090 | 8976 | 1.24 | 90.08 | 101.4 | 0.89 | 0.46 | 0.58 | 0.79 | 1.01 | 1.65 | 1.64 |
| 10-fasted | 20723 | 17716 | 1.17 | 183.0 | 237 | 0.77 | 0.50 | 0.58 | 0.86 | 1.00 | 1.71 | 1.70 |
| 25-fasted | 54595 | 40505 | 1.35 | 466.0 | 413.2 | 1.13 | 0.47 | 0.61 | 0.76 | 1.01 | 1.52 | 1.51 |
| 50-fasted | 110038 | 90382 | 1.22 | 912.3 | 980.7 | 0.93 | 0.47 | 0.58 | 0.80 | 1.01 | 1.26 | 1.25 |
| 50-fed | 106678 | 92906 | 1.15 | 792.6 | 833.6 | 0.95 | 0.48 | 0.57 | 0.85 | 1.01 | 1.37 | 1.35 |
| 100-fasted | 232684 | 217928 | 1.07 | 1823 | 2127 | 0.86 | 0.44 | 0.50 | 0.88 | 1.01 | 1.07 | 1.07 |
| 150-fasted | 325589 | 269346 | 1.21 | 2802 | 3327 | 0.84 | 0.47 | 0.66 | 0.72 | 1.01 | 1.13 | 1.12 |
| Multiple dose | 54888 | 48182 | 1.14 | 628.3 | 532.7 | 1.18 | 0.65 | 0.55 | 1.17 | 1.01 | 1.08 | 1.07 |

Table S3 Comparison of the predicted and observed PK parameters based on the second method

| Dose  (mg) | AUC  (ng/mL×h) | | | C_max_  (ng/mL) | | | CL  (L/h) | | | V_Z_/F  (L/kg) | | |
| --- | --- | --- | --- | --- | --- | --- | --- | --- | --- | --- | --- | --- |
|  | Pred | Obs | Ratio | Pred | Obs | Ratio | Pred | Obs | Ratio | Pred | Obs | Ratio |
| 5-fasted | 11137 | 8976 | 1.24 | 89.34 | 101.4 | 0.88 | 0.46 | 0.58 | 0.79 | 1.01 | 1.65 | 1.64 |
| 10-fasted | 20805 | 17716 | 1.17 | 180.4 | 237.0 | 0.76 | 0.49 | 0.58 | 0.86 | 1.00 | 1.71 | 1.70 |
| 25-fasted | 54061 | 40505 | 1.33 | 451.2 | 413.2 | 1.09 | 0.47 | 0.61 | 0.77 | 1.01 | 1.52 | 1.51 |
| 50-fasted | 109428 | 90382 | 1.21 | 861.5 | 980.7 | 0.88 | 0.47 | 0.58 | 0.81 | 1.01 | 1.26 | 1.25 |
| 50-fed | 110503 | 92906 | 1.19 | 812.9 | 833.6 | 0.98 | 0.46 | 0.57 | 0.82 | 1.01 | 1.37 | 1.35 |
| 100-fasted | 225356 | 217928 | 1.03 | 1583 | 2127 | 0.74 | 0.46 | 0.50 | 0.91 | 1.01 | 1.07 | 1.07 |
| 150-fasted | 289154 | 269346 | 1.07 | 2107 | 3327 | 0.63 | 0.55 | 0.66 | 0.84 | 1.01 | 1.13 | 1.12 |
| Multiple dose | 55046 | 48182 | 1.14 | 613.2 | 532.7 | 1.15 | 0.65 | 0.55 | 1.17 | 1.01 | 1.08 | 1.07 |
